# Hidden Burden of ICU: Patient-Perceived Stressors After Cardiothorasic Surgery

**DOI:** 10.3390/jcm15062276

**Published:** 2026-03-17

**Authors:** Karolina Ozdowska, Katarzyna Lewandowska, Katarzyna Czyż-Szypenbejl, Kazimiera Hebel, Aleksandra Steliga, Wioletta Mędrzycka-Dąbrowska

**Affiliations:** 1 Diaverum Dialysis Center, 80 Ceglowska Street, 01-809 Warsaw, Poland; ozdowska696@gmail.com; 2 Department of Anesthesiology and Intensive Care Nursing, Medical University of Gdansk, 7 Debinki Street, 80-211 Gdansk, Poland; kalewandowska@gumed.edu.pl (K.L.); wioletta.medrzycka@gumed.edu.pl (W.M.-D.); 3 Institute of Health Sciences, Pomeranian University of Slupsk, 64 Bohaterow Westerplatte St., 76-200 Slupsk, Poland; kazimiera.hebel@upsl.edu.pl (K.H.); aleksandra.steliga@upsl.edu.pl (A.S.)

**Keywords:** ICU, patient-centered care, nursing care

## Abstract

**Background/Objectives:** Patients after cardiac surgery admitted to the intensive care unit (ICU) are exposed to environmental, procedural, and psychological stressors that may affect comfort and recovery. This study aimed to assess perceived ICU stressors in postoperative cardiac surgery patients, identify the most and least distressing factors, and examine associations between stressor intensity and selected clinical and organizational variables. **Methods:** A single-center cross-sectional survey was conducted in an ICU in Poland (January 2024–February 2024). Adult patients after cardiac surgery who provided informed consent and had no cognitive impairment were included; cognitive status was screened using the Montreal Cognitive Assessment (MoCA). Perceived stressors were measured using the Intensive Care Unit Environmental Stressor Scale (ICUESS; 40 items; 4-point Likert scale). **Results:** The highest-rated stressors were sleep problems (M = 2.30; SD = 0.86) and hearing heart monitor alarms (M = 2.16; SD = 0.82). The lowest-rated stressors were not knowing what day it was (M = 1.46; SD = 0.54) and nurses not introducing themselves (M = 1.50; SD = 0.54). Longer respiratory support and higher pain intensity were associated with higher stressor ratings for multiple ICUESS items, whereas age showed no significant association. Higher room occupancy was linked to higher perceived stress related to environmental disturbances. ICU length of stay showed only limited item-level associations. **Conclusions:** Postoperative cardiac surgery patients experience a multifactorial burden of ICU stressors, with sleep disruption and alarm-related noise among the most distressing. Prioritizing modifiable environmental factors, symptom control (particularly pain), and patient-centered communication may help reduce perceived stress, especially in shared-room settings and among patients requiring longer respiratory support.

## 1. Introduction

From the moment patients are admitted to an Intensive Care Unit (ICU), they are exposed to a high technology environment and multiple potentially distressing stimuli. Continuous monitoring, frequent therapeutic interventions, and sensory overload may disrupt sleep and contribute to pain, dyspnea, anxiety, and frightening experiences such as hallucinations, particularly when patients are unable to communicate their needs effectively [1,2,3].

In this context, stressors can be understood as internal or external stimuli that trigger a stress response and require somatic, psychological, or behavioral adaptation [4]. The concept of the general adaptation syndrome, described by H. Selye, highlights physiological mechanisms involved in responding to detrimental triggers [5]. In the ICU, stressors may include noise coming from various equipment, unpleasant odors, bright light, sleep disruption, lack of control, restrictions on visits from relatives, and pain. Identifying and mitigating these stressors is clinically important, as persistent distress may be associated with adverse psychological outcomes, including delirium and post-traumatic stress symptoms in patients and their relatives [6,7].

Supporting critically ill patients in coping with ICU-related stressors is a responsibility shared by the interdisciplinary team. Because many elements of the ICU environment are modifiable, systematic recognition of unit-specific stressors may facilitate a more therapeutic milieu and holistic, patient-centered care [8]. Nurses play a central role in identifying and addressing distressing factors, given their continuous bedside presence and close monitoring patients’ vital signs and response to nursing interventions. However, the perception of stressors may differ between patients and caregivers; nurses’ assessments can be influenced by professional training and prior clinical experience [8].

Despite the growing use of percutaneous and minimally invasive techniques, open cardiac surgery remains the standard of care in selected cases. Following procedures performed via thoracotomy or sternotomy, patients are routinely admitted to the ICU for postoperative stabilization of respiratory and circulatory functions. This setting involves continuous advanced, and often invasive, monitoring and treatment methods, as well as frequent alarms and interventions, all of which may intensify patient distress. Therefore, identifying stressors within a given unit and implementing targeted interventions may help reduce the burden of the ICU environment on postoperative cardiac surgery patients [9].

The aim of this study was to assess perceived ICU-related stressors among patients after cardiac surgery and to examine their associations with selected clinical and environmental factors, including patient room occupancy, duration of respiratory support, age, ICU length of stay, and pain intensity.

## 2. Materials and Methods

### 2.1. Study Design

We conducted a cross-sectional survey. The study was conducted in the postoperative intensive care unit within the Department of Anesthesiology and Intensive Care in a university clinical hospital in Poland, with permission from the hospital director. The study protocol complied with the principles of the Declaration of Helsinki and was approved by the Independent Bioethics Committee for Scientific Research at the Medical University of Gdansk (approval No. KB/573/2023 and date of approval 20 October 2023).

The manuscript is reported in accordance with the Strengthening Reporting of Observational Studies in Epidemiology (STROBE) statement.

### 2.2. Research Questions

1. Which ICU-related stressors are perceived as the most and the least distressing patients after cardiac surgery?
2. Is the number of patients per room associated with the perception of ICU-related stressors?
3. Is the duration of respiratory support (e.g., mechanical ventilation) associated with the intensity of any perceived stressors?
4. Is age associated with the perception of ICU-related stressors?
5. Is the ICU length of stay associated with the intensity of any perceived stressors?
6. Is pain intensity associated with the perception of ICU-related stressors?

### 2.3. ICU Environment

The study was conducted in the intensive care unit (ICU) of a clinical hospital in Poland. The nurse-to-patient ratio was 1:2 or 1:1, depending on patient acuity. The unit has an open-space layout with a shared patient area, except for three isolation rooms. The ICU comprises 10 beds, with adequate spacing between beds and privacy curtains to support patient privacy and dignity. Natural daylight is available.

Each bed space is equipped for continuous monitoring of vital signs and includes an adjustable ICU bed with a pressure-redistribution mattress for pressure injury (PI) prevention. Vital signs are monitored continuously and documented hourly. Standard monitoring includes a 5-lead electrocardiogram (ECG), pulse oximetry (finger or earlobe sensor), and pressure transducers for continuous invasive arterial blood pressure and central venous pressure monitoring, as well as end-tidal carbon dioxide levels. Nursing care is provided at the bedside, and continuous observation is ensured from the central nursing station.

Morning hygiene care is routinely performed after rounds, and PI prevention measures are implemented throughout the day according to individual needs. Physiotherapy is provided between 8:00 a.m. and 3:00 p.m. Early mobilization protocols are implemented if the patient is hemodynamically stable. Many point of care testing is available (i.e., portable radiography; arterial blood gas (ABG); ultrasound and echocardiography). ABG analysis is routinely performed every 6 h or more frequently if clinically indicated. Other laboratory tests are performed daily at 6:00 a.m.

### 2.4. Inclusion/Exclusion Criteria

Adult patients admitted to the ICU after cardiac surgery were eligible for the study if they provided informed consent and had no evidence of cognitive impairment. In total, 69 patients met the eligibility criteria; 19 declined to participate. Patients with cognitive impairment, delirium, and those receiving mechanical ventilation at the time of assessment were excluded.

### 2.5. Questionnaire and Variables

Cognitive status was screened using the Montreal Cognitive Assessment (MoCA) [10], a brief tool developed to detect mild cognitive impairment. MoCA assesses multiple cognitive domains (including attention, executive functions, memory, language, visuospatial abilities, abstraction, calculation, and orientation). The test takes approximately 10 min to administer and yields a total score ranging from 0 to 30 points; a cut-off of ≥26 is commonly used to indicate normal cognitive performance. The assessment was administered by trained staff in accordance with MoCA requirements.

Perceived ICU-related stressors were measured with the Intensive Care Unit Environmental Stressor Scale (ICUESS) [11], a Likert-type questionnaire comprising 40 ICU stressors rated by patients. Each item is scored from 1 (“no stress”) to 4 (“very high stress”). Permission to use the instrument was obtained from the original author(s). The Polish version was developed using a forward–backward translation procedure: (1) forward translation by two independent experts followed by reconciliation into a single Polish version; (2) backward translation by an independent translator; and (3) expert review by three faculty members with clinical expertise in intensive care to assess content validity.

Internal consistency of the ICUESS in the present study was evaluated using Cronbach’s α and indicated very good reliability (α = 0.952) for the 40-item scale.

Demographic and clinical variables collected included sex, age, ICU length of stay, type of surgery, duration of respiratory support, history of prior hospitalization, pain intensity assessed with the Numeric Rating Scale (NRS), and the number of patients in the room at the time of assessment.

### 2.6. Setting

Data collection was carried out between January 2024 and February 2024. Prior to participation, all patients received written and verbal information regarding the purpose and procedures of the study and provided written informed consent. Cognitive status was screened using the MoCA. Patients with no evidence of cognitive impairment (MoCA ≥ 26 points) were subsequently asked to complete ICUESS. The assessment was performed within the first 24 h after extubation. The researcher was present during questionnaire completion to provide clarification if needed, without influencing patients’ responses. After questionnaires were collected, data was coded and analyzed.

### 2.7. Statistical Analysis

Statistical analyses were performed using IBM SPSS Statistics (version 30). The internal consistency of the ICUESS was evaluated using Cronbach’s α. Descriptive statistics were used to summarize patient characteristics and stressor ratings (including distributions of responses for individual items). Group comparisons were conducted using the Mann–Whitney U test. Associations between variables were examined using Spearman’s rank correlation coefficient (rho). A two-sided significance level of *p* < 0.05 was adopted. Because correlations were examined between multiple ICUESS items and several clinical and environmental variables, these analyses were considered exploratory. Therefore, no formal adjustment for multiple comparisons was applied

## 3. Results

### 3.1. Demographical Data

The study group comprised 50 patients. Most participants were men (n = 43, 86.0%), while women accounted for 14.0% (n = 7) (Table 1). Participants’ age ranged from 22 to 86 years (M = 66.66, SD ± 11.60). The primary reason for admission to the cardiac ICU was postoperative care after cardiac surgery (n = 49), whereas one patient was admitted for another indication.

Coronary artery bypass grafting (CABG) was the most common procedure, followed by aortic valve replacement and mitral valve repair. The ICU length of stay ranged from 1 to 24 days (M = 2.98, SD ± 3.59). The mean duration of mechanical ventilation was 13.76 ± 15.85 h. The number of previous hospitalizations ranged from 0 to 17 per patient (M = 1.78, SD ± 2.97). The mean MoCA score was M = 27.64 (SD ± 1.10).

### 3.2. Assessment of the Intensity of Stressors

The results indicated that the highest rated stressors were sleep problems (M = 2.30; SD = 0.86) and hearing the heart monitor alarms (M = 2.16; SD = 0.82) (Table 2.), while the lowest rated were not knowing what day it was (M = 1.46; SD = 0.54) and the fact that the nurses did not introduce themselves (M = 1.50; SD = 0.54). The two lowest-rated stressors were the only items with a median of 1.00, indicating that more than half of the surveyed patients considered them not stressful. In contrast, the highest-rated stressors were sleep problems (M = 2.30) and hearing the heart monitor alarm (M = 2.16) on a scale ranging from 1 to 4, suggesting that these factors were perceived as relatively more distressing compared with other items.

### 3.3. Factors Determining the Severity of Stressors

With increasing duration of respiratory therapy, the level of perceived stress rose with respect to most ICU-related factors, whereas no association was found with patient age (Table 3).

The greater the number of patients in the room, the higher the level of perceived stress related to the environmental stressors in this particular ward (hearing unfamiliar and strange sounds, being woken up by nurses, smelling strange odors, being disturbed by others, feeling that nurses were working too fast, hearing other patients groaning, hearing the sounds of the heart rate monitor alarm, feeling that nurses were spending more time on the monitors than on the patients, being constantly checked by nurses and doctors, being surrounded by unfamiliar machines, having one’s head and not knowing the doctor who was supervising the care) and related to being hospitalized in the ICU (being thirsty, the bed and/or pillow being uncomfortable, having to look at the ceiling, not being able to move hands or arms due to IV drips, having IV drips above one’s head).

Stressors that were not dependent on the time of respiratory therapy included: sleep problems, set temperature in the room, unfamiliar and strange ambient sounds, lack of self-control, lack of orientation in place and time, feeling disturbed by others, lack of mobility due to being connected to the machine, and having the lights on all the time.

Women considered pain, thirst, and the feeling that nurses spent more time on monitors than on patients to be significantly more stressful than men. The stress associated with checking blood pressure several times a day increased with the number of days spent at the ICU. In the case of the remaining variables, no statistical significance relationships were found, which meant that the change in the length of stay did not correlate with the change in the level of perceived stress related to factors occurring in the ICU.

As the pain intensity increased, the level of stress increased due to the occurrence of pain, thirst, hearing unfamiliar and strange sounds, the feeling that nurses were working in too much of a hurry, hearing the groans of other patients, not being able to see family and friends for several minutes a day, and not knowing the doctor supervising the care.

Statistically significant differences between first-time hospitalized and re-hospitalized patients were observed for several stressors, including being pricked with a needle, not knowing what day it was, IV drips hanging over the patient’s head, and the nurse not introducing herself. The complete set of comparisons is presented in Supplementary Table S1.

## 4. Discussion

The findings of this study are consistent with previous research and align with the study objectives [2,3,12,13]. Specifically, we aimed to assess the intensity of perceived ICU stressors in postoperative cardiac surgery patients, identify the most and least distressing factors, and explore whether stressor perception was associated with selected clinical and organizational variables (e.g., duration of respiratory support, pain intensity, room occupancy, and ICU length of stay). Patients hospitalized in the intensive care unit are exposed to multiple stressors that should be systematically identified and addressed. ICU-related stressors are commonly grouped into three domains: physical, psychological, and environmental. In our study, physical stressors were perceived as the most stressful among the top 10 items reported in ICU, which is consistent with earlier work [3,12,13].

Over the last 30 years, studies have consistently described the most common ICU stressors. Across settings, authors have generally agreed that key stressors include pain, thirst, the presence of tubes, sleep disturbance, and missing loved ones [3,14,15]. In our study, the five most stressful items were: inability to sleep, hearing the heart monitor alarm, hearing other patients moaning, feeling pain, and missing one’s husband/wife.

Sleep disturbance is multifactorial, and ICU noise is a well-recognized contributor. Monitoring devices (e.g., heart monitors) equipped with warning systems are widely used in ICU environments. According to World Health Organization guidelines, sound levels should not exceed 35 dB in most patient rooms [16]; however, several studies have reported that noise levels frequently exceed 50 dBA [17,18,19,20]. Alarm signals can be perceived as threatening, as they engage in neural pathways similar to those activated by human screams. Importantly, a substantial proportion of ICU alarms are false alarms [21], highlighting the need for accurate alarm management and optimization.

In addition to device alarms, noise generated by staff and other patients may increase distress. In our study, “hearing other patients moaning” ranked among the five highest-scored stressors. A plausible explanation is the architectural design of the studied ICU; evidence suggests that higher patient density within rooms is associated with greater stress, particularly with respect to environmental stressors. Because ICU environments, organizational structures, and clinical practices may differ across institutions, the perception of ICU-related stressors may also vary between settings. Therefore, conducting similar assessments using standardized tools such as the ICU Environmental Stressor Scale (ICUESS) in other ICUs may help identify locally relevant stressors and guide targeted improvements in patient-centered care.

Disruptions in relationships and the inability to perform social roles may also contribute to anxiety. Our study found that missing one’s partner was highly stressful, consistent with previous research [22]. In the studied ICU, visiting hours are limited to 1.5 h daily (15:30–17:00), which may further exacerbate stress. Strategies such as liberalizing visitation policies, encouraging patient diaries, and improving communication with loved ones may mitigate some adverse effects of the ICU environment [23,24]. It is also worth emphasizing that the families of patients hospitalized in the ICU also experience high levels of stress and anxiety [25].

In our cohort of postoperative cardiac surgery patients, the duration of respiratory support emerged as a key factor associated with the intensity of perceived ICU stressors. Although the mean duration of respiratory therapy/mechanical ventilation was relatively short (13.76 ± 15.85 h), longer respiratory support was accompanied by higher stress ratings across most ICUESS items. This pattern suggests that even in the early postoperative period, prolonged exposure to respiratory support may coincide with a greater burden of distressing experiences, likely reflecting more intensive monitoring and bedside interventions, reduced autonomy, communication barriers, and greater symptom burden. These findings are supported by Takashima et al., who assessed stressful experiences among mechanically ventilated ICU patients ventilated for more than 12 h and reported higher stress levels with longer intubation duration [26]. Moreover, recent qualitative research (2024) indicates that mechanically ventilated patients frequently experience substantial physical and psychological suffering [27], and that communication difficulties and the inability to express needs and emotions further intensify stress and frustration [28].

In our study, patient age was not associated with the intensity of perceived ICU stressors. This suggests that, in electively admitted postoperative cardiac surgery patients, stressor burden may be driven primarily by early postoperative symptoms and routine aspects of intensive care rather than by age-related differences in stress appraisal. Similar findings have been reported in ICUESS-based studies where mean stressor scores did not differ significantly across age groups [29]. However, the evidence is not entirely consistent. Mollaoğlu et al. reported that ICU stressor perception may vary with age, with older patients describing higher ICUESS scores [30]. Taken together, these divergent findings imply that the association between age and perceived ICU stressors is likely context dependent. In our cohort, the planned nature of ICU admission after cardiac surgery and the exclusion of patients with delirium or cognitive impairment may have reduced age-related variability in how stressors were interpreted and reported, resulting in a more homogeneous stress profile.

Jennerich et al. reported that emergent (unplanned) ICU admissions are associated with higher perceived stress compared with planned hospitalizations [31]. This observation is also consistent with Takashima’s report that patients with no previous history of illness who are admitted to the ICU in emergency situations experience significantly higher stress [26]. In our study, most patients were admitted to the ICU following scheduled cardiac surgery; therefore, they could anticipate the postoperative ICU stay and receive preoperative preparation. Such preparation may include psychological support and structured information about the ICU environment and routine care, which may help patients cope with selected stressors during the postoperative period. Notably, ICU length of stay was not associated with the overall intensity of perceived stressors in our cohort, consistent with recent ICUESS-based studies published in 2021 and 2022 that also did not demonstrate a significant relationship between ICU stay duration and perceived stressor burden [32,33].

Many studies have identified pain as one of the top five ICU stressors [2,30,34]. Although pain was not the most stressful experience in our study, there is evidence that pain can be the most traumatic experience in certain settings [12,30,35]. Even when appropriate practices are implemented, pain cannot always be avoided in ICU patients. Pain may result from procedures or prolonged immobility (e.g., muscle or joint pain). Therefore, pain monitoring is essential, particularly in sedated or mechanically ventilated (non-verbal) patients, using validated behavioral tools such as the Critical-Care Pain Observation Tool (CPOT) or the Behavioral Pain Scale (BPS) [36]. In our experience, the use of such scales remains limited in some departments, and nursing staff are not always adequately trained in their application. It should also be noted that the perception and intensity of ICU-related stressors may differ across surgical populations and institutional settings. Therefore, future studies should include comparative analyses of patients admitted to the ICU after different types of surgical procedures. Such assessments may also support the identification of modifiable environmental and organizational factors and guide targeted quality improvement initiatives, followed by subsequent evaluations to determine whether these interventions reduce the burden of patient-perceived stressors.

## 5. Conclusions

The highest-rated ICU stressors were sleep problems and hearing heart monitor alarms, whereas the lowest-rated stressors were not knowing what day it was and nurses not introducing themselves. A higher number of patients per room was associated with greater perceived stress, particularly environmental stressors and disturbances. Longer duration of respiratory support was associated with higher stress ratings for most assessed stressors. Patient age was not significantly associated with perceived stressor intensity. ICU’s length of stay was not related to the intensity of most stressors (with only isolated item-level associations). Higher pain intensity was associated with higher perceived stress and greater intensity of selected concurrent stressors.

## 6. Implication to Clinical Practice

The findings provide several actionable implications for ICU practice, particularly nursing care. First, modifiable environmental factors should be considered central targets of patient-centered care in open-plan ICUs. Interventions such as optimizing alarm settings, reducing nighttime noise and light, minimizing unnecessary awakenings, and ensuring privacy through consistent use of curtains may lower perceived stress. Second, symptom-focused strategies—especially pain and thirst management—should be prioritized, as they may influence the perception of multiple stressors. Third, clear communication practices (introducing staff, explaining procedures, identifying the responsible physician, and providing basic orientation) may reduce anxiety and improve patients’ sense of control, particularly among first-time hospitalized patients. These approaches may also contribute indirectly to reducing longer-term psychological sequelae following critical illness, including features described within post-intensive care syndrome.

## 7. Study Limitations

This study has several limitations that should be considered when interpreting the results. First, the sample size was relatively small and derived from a single intensive care unit, which may limit the generalizability of the findings to other ICU settings or patient populations. Second, the study group was characterized by a marked predominance of male participants, which reflects the epidemiology of cardiac surgery populations but limits the reliability of sex-based comparisons and the interpretation of gender-related differences [37,38]. Third, the cross-sectional design of the study allows only the identification of associations and does not permit conclusions regarding causal relationships between ICU-related stressors and the examined clinical or environmental variables. In addition, the analysis included multiple correlation tests between individual ICUESS items and several variables. Although these analyses were conducted to explore potential patterns of association, testing numerous correlations may increase the risk of Type I error. Therefore, the observed associations should be interpreted with caution and considered exploratory rather than confirmatory.

The study included a small sample size due to the severity of the patients’ condition (cognitive impairment, inability to complete the questionnaire). Therefore, the results cannot be extrapolated to the entire ICU patient population. It is difficult to compare the female and male groups, as the study included significantly fewer women. It is impossible to compare the nurses’ perspectives with those of the patients, as the study did not include nursing staff.

## Figures and Tables

**Table 1:** Demographic and clinical characteristics of the study group (n = 50).

Variable	M ± SD/n (%)
Sex	
Female	7 (14.0%)
Male	43 (86.0%)
Age (years)	66.66 ± 11.60
ICU length of stay (days)	2.98 ± 3.59
Type of surgery	
Coronary artery bypass grafting (CABG)	16 (32.0%)
Mitral valve repair	10 (20.0%)
Aortic valve repair	8 (16.0%)
Tricuspid valve repair	1 (2.0%)
Mitral valve replacement	8 (16.0%)
Aortic valve replacement	10 (20.0%)
Other	1 (2.0%)
Duration of mechanical ventilation (hours), mean ± SD	13.76 ± 15.85
Previous hospitalization	
No	26 (52.0%)
Yes	24 (48.0%)
Number of previous hospitalizations, mean ± SD	3.71 ± 3.37
Number of patients per room	
1	3 (6.0%)
2	14 (28.0%)
3	2 (4.0%)
4	3 (6.0%)
5	20 (40.0%)
6	8 (16.0%)
Pain intensity (NRS), mean ± SD	1.18 ± 1.78
MoCA score (points), mean ± SD	27.64 ± 1.10

ICU—intensive care unit; CABG—coronary artery bypass grafting; NRS—Numeric Rating Scale; MoCA—Montreal Cognitive Assessment; SD—standard deviation, M—mean.

**Table 2:** Stressor intensity ratings (ICUESS items).

Stressor	M	Me	SD	Skewness	Kurtosis	Min	Max
I feel pain	2.12	2.00	0.80	0.28	−0.36	1.00	4.00
I have trouble sleeping	2.30	2.00	0.86	0.16	−0.57	1.00	4.00
I have tubes in my nose/mouth	1.86	2.00	0.83	0.71	−0.04	1.00	4.00
I am being pricked with a needle	1.74	2.00	0.69	0.40	−0.84	1.00	3.00
Being thirsty	2.04	2.00	0.75	−0.07	−1.21	1.00	3.00
My bed and/or pillow are uncomfortable	1.94	2.00	0.79	0.62	0.18	1.00	4.00
I have to breathe oxygen	1.80	2.00	0.73	0.33	−1.02	1.00	3.00
The room is too hot or too cold	1.86	2.00	0.73	0.55	0.21	1.00	4.00
I hear unfamiliar and strange sounds	2.02	2.00	0.71	0.67	1.07	1.00	4.00
A nurse woke me up	1.78	2.00	0.65	0.24	−0.62	1.00	3.00
I smell strange odors	1.78	2.00	0.74	0.70	0.31	1.00	4.00
I have no control over myself	1.80	2.00	0.70	0.67	0.73	1.00	4.00
No one discussed my treatment with me	1.86	2.00	0.81	0.99	1.07	1.00	4.00
I do not know when a procedure will be performed on me	2.06	2.00	0.74	0.53	0.50	1.00	4.00
I have no privacy	1.96	2.00	0.88	0.64	−0.23	1.00	4.00
Someone is disturbing me	1.76	2.00	0.66	0.29	−0.68	1.00	3.00
I do not know where I am	1.70	2.00	0.79	1.12	1.16	1.00	4.00
I do not know what time it is	1.64	2.00	0.66	0.55	−0.64	1.00	3.00
I feel that nurses are working in too much of a hurry	1.82	2.00	0.66	0.21	−0.67	1.00	3.00
I do not know what day it is	1.46	1.00	0.54	0.56	−0.89	1.00	3.00
I am forced to look at the ceiling	1.66	2.00	0.59	0.26	−0.61	1.00	3.00
I cannot move because I am connected to equipment	2.06	2.00	0.79	0.66	0.46	1.00	4.00
I cannot move my hands or arms because I am connected to an IV drip	1.80	2.00	0.73	0.66	0.35	1.00	4.00
The lights are on all the time	1.98	2.00	0.71	0.38	0.13	1.00	4.00
Hearing other patients groaning	2.12	2.00	0.85	0.18	−0.77	1.00	4.00
I hear the heart monitor alarm	2.16	2.00	0.82	0.16	−0.59	1.00	4.00
The medical staff use terms I do not understand	1.84	2.00	0.62	0.11	−0.37	1.00	3.00
I feel that nurses spend more time on the monitors than on me	1.82	2.00	0.69	0.64	0.81	1.00	4.00
Nurses are constantly moving around my bed	1.74	2.00	0.60	0.16	−0.46	1.00	3.00
I hear noise and machine alarms	2.06	2.00	0.74	0.22	−0.31	1.00	4.00
I am constantly examined by nurses and doctors	1.74	2.00	0.66	0.34	−0.71	1.00	3.00
I hear the telephone ringing	1.74	2.00	0.69	0.40	−0.84	1.00	3.00
I am surrounded by unfamiliar machines	1.98	2.00	0.82	0.27	−0.88	1.00	4.00
My blood pressure is checked several times a day	1.94	2.00	0.93	0.75	−0.26	1.00	4.00
IV drips are hanging over my head	1.94	2.00	0.77	0.10	−1.27	1.00	3.00
Seeing family and friends only a few minutes a day	1.84	2.00	0.74	0.58	0.14	1.00	4.00
Nurses and doctors talk too loudly	1.68	2.00	0.62	0.33	−0.60	1.00	3.00
I miss my husband or wife	2.10	2.00	0.84	0.02	−1.11	1.00	4.00
I do not know the doctor in charge of my care	1.88	2.00	0.75	0.51	−0.04	1.00	4.00
The nurse does not introduce herself to me	1.50	1.00	0.54	0.40	−1.05	1.00	3.00

M—mean; Me—median; SD—standard deviation; Min—minimum; Max—maximum.

**Table 3:** Factors determining the severity of stressors.

Variable	Number of Patients Per Room	Duration of Mechanical Ventilation	Age	ICU Length of Stay	Pain Intensity
I feel pain	0.16	0.35 *	0.06	0.18	0.35 *
I have trouble sleeping	0.03	0.27	0.06	−0.07	0.14
I have tubes in my nose/mouth	0.17	0.43 **	0.01	−0.01	0.09
I am being pricked with a needle	0.08	0.4 3 **	0.09	−0.03	−0.09
Being thirsty	0.33 *	0.44 ***	0.14	0.01	0.32 *
My bed and/or pillow are uncomfortable	0.32 *	0.33 *	−0.06	−0.23	0.09
I have to breathe oxygen	0.24	0.43 **	−0.14	0.02	0.03
The room is too hot or too cold	0.24	0.25	−0.01	0.14	0.23
I hear unfamiliar and strange sounds	0.30 *	0.28	0.10	0.19	0.39 **
A nurse woke me up	0.30 *	0.45 ***	−0.09	0.07	0.12
I smell strange odors	0.36 **	0.54 ***	−0.08	0.01	0.15
I have no control over myself	0.02	0.18	−0.13	0.20	0.17
No one discussed my treatment with me	0.04	0.30 *	−0.22	0.02	0.19
I do not know when a procedure will be performed on me	0.20	0.37 **	−0.12	0.11	0.13
I have no privacy	0.21	0.35 *	−0.04	−0.16	0.21
Someone is disturbing me	0.32 *	0.19	−0.04	0.27	0.07
I do not know where I am	0.10	0.16	−0.06	0.09	0.08
I do not know what time it is	0.05	0.21	−0.04	−0.07	0.04
I feel that nurses are working in too much of a hurry	0.42 **	0.62 ***	−0.13	0.05	0.28 *
I do not know what day it is	0.18	0.29 *	−0.05	−0.16	0.12
I am forced to look at the ceiling	0.33 *	0.58 ***	−0.08	0.24	0.13
I cannot move because I am connected to equipment	0.10	0.19	0.03	−0.10	0.18
I cannot move my hands or arms because I am connected to an IV drip	0.32 *	0.58 ***	−0.10	0.08	0.16
The lights are on all the time	−0.02	0.19	0.26	0.24	0.20
Hearing other patients groaning	0.31 *	0.33 *	0.22	0.03	0.43 **
I hear the heart monitor alarm	0.28 *	0.48 ***	0.13	−0.04	0.17
The medical staff use terms I do not understand	0.25	0.45 ***	−0.04	−0.02	0.25
I feel that nurses spend more time on the monitors than on me	0.35 *	0.51 ***	−0.01	−0.01	0.18
Nurses are constantly moving around my bed	0.27	0.54 ***	−0.07	0.13	0.13
I hear noise and machine alarms	0.19	0.52 ***	<0.01	0.24	0.26
I am constantly examined by nurses and doctors	0.33 *	0.54 ***	−0.03	0.06	0.14
I hear the telephone ringing	0.08	0.33 *	0.06	0.15	0.16
I am surrounded by unfamiliar machines	0.29 *	0.60 ***	0.08	0.11	0.16
My blood pressure is checked several times a day	0.28	0.48 ***	−0.03	0.28 *	0.08
IV drips are hanging over my head	0.47 ***	0.46 ***	0.04	<0.01	0.19
Seeing family and friends only a few minutes a day	0.22	0.39 **	0.11	0.01	0.29 *
Nurses and doctors talk too loudly	0.28	0.24	−0.06	0.19	−0.01
I miss my husband or wife	0.25	0.30 *	−0.12	0.03	0.05
I do not know the doctor in charge of my care	0.28 *	0.47 ***	−0.10	0.15	0.39 **
The nurse does not introduce herself to me	0.22	0.54 ***	0.10	0.12	0.09

Values are Spearman’s rho correlation coefficients. * *p* < 0.05; ** *p* < 0.01; *** *p* < 0.001.

## Data Availability

The data that support the findings of this study are available from the main author (K.O.) upon reasonable request.

## Supplementary Materials

The following supporting information can be downloaded at: https://www.mdpi.com/article/10.3390/jcm15062276/s1, Table S1: Comparison of patients hospitalized for the first time with patients hospitalized for a subsequent time in terms of the assessment of individual stressors.
