# Supplementary material for: Hidden Burden of ICU: Patient-Perceived Stressors After Cardiothorasic Surgery
Source: J Clin Med. 2026 Mar 17;15(6):2276. doi: 10.3390/jcm15062276 (PMC13027096; doi:10.3390/jcm15062276)
Supplement: Supplementary file 1 [file jcm-15-02276-s001.zip › jcm-4179017-supplementary.pdf]

Table S1. *Comparison of patients hospitalized for the first time with patients hospitalized for a subsequent time in terms of the assessment of individual stressors.*

	Re-hospitalized patients (n = 24)			First-time hospitalized patients (n = 26)				
Stressor	Mean rank	M	SD	Mean rank	M	SD	Z	η^2
I feel pain	25.69	2.13	0.95	25.33	2.12	0.65	-0.09	<0.01
I have trouble sleeping	28.35	2.46	0.88	22.87	2.15	0.83	-1.41	0.04
I have tubes in my nose/mouth	25.48	1.92	1.02	25.52	1.81	0.63	-0.01	<0.01
I am being pricked with a needle	20.44	1.50	0.72	30.17	1.96	0.60	-2.58**	0.14
Being thirsty	24.06	1.96	0.81	26.83	2.12	0.71	-0.72	0.01
My bed and/or pillow are uncomfortable	24.83	1.92	0.88	26.12	1.96	0.72	-0.34	<0.01
I have to breathe oxygen	21.77	1.63	0.82	28.94	1.96	0.60	-1.88	0.07
The room is too hot or too cold	25.94	1.88	0.74	25.10	1.85	0.73	-0.22	<0.01
I hear unfamiliar and strange sounds	23.56	1.96	0.86	27.29	2.08	0.56	-1.04	0.02
A nurse woke me up	22.75	1.67	0.76	28.04	1.88	0.52	-1.43	0.04
I smell strange odors	23.40	1.71	0.86	27.44	1.85	0.61	-1.07	0.02
I have no control over myself	25.54	1.83	0.82	25.46	1.77	0.59	-0.02	<0.01
No one discussed my treatment with me	25.73	1.96	1.04	25.29	1.77	0.51	-0.12	<0.01
I do not know when a procedure will be performed on me	25.29	2.08	0.97	25.69	2.04	0.45	-0.11	<0.01
I have no privacy	22.75	1.83	1.01	28.04	2.08	0.74	-1.37	0.04
Someone is disturbing me	25.00	1.75	0.74	25.96	1.77	0.59	-0.26	<0.01
I do not know where I am	23.90	1.67	0.92	26.98	1.73	0.67	-0.82	0.01
I do not know what time it is	21.94	1.46	0.59	28.79	1.81	0.69	-1.84	0.07
I feel that nurses are working in too much of a hurry	22.98	1.71	0.75	27.83	1.92	0.56	-1.31	0.03
I do not know what day it is	21.65	1.29	0.46	29.06	1.62	0.57	-2.07*	0.09
I am forced to look at the ceiling	22.52	1.54	0.66	28.25	1.77	0.51	-1.57	0.05
I cannot move because I am connected to equipment	25.81	2.13	0.95	25.21	2.00	0.63	-0.16	<0.01
I cannot move my hands or arms because I am connected to an IV drip	22.98	1.71	0.86	27.83	1.88	0.59	-1.29	0.03
The lights are on all the time	25.44	2.00	0.88	25.56	1.96	0.53	-0.03	<0.01
Hearing other patients groaning	26.29	2.17	0.87	24.77	2.08	0.84	-0.39	<0.01
I hear the heart monitor alarm	21.98	1.96	0.86	28.75	2.35	0.75	-1.75	0.06
The medical staff use terms I do not understand	23.50	1.75	0.68	27.35	1.92	0.56	-1.07	0.02
I feel that nurses spend more time on the monitors than on me	23.40	1.71	0.69	27.44	1.92	0.69	-1.10	0.02
Pielęgniarki cały czas krzątają się wokół łóżka	23.79	1.67	0.64	27.08	1.81	0.57	-0.91	0.02
I hear noise and machine alarms	24.13	2.00	0.83	26.77	2.12	0.65	-0.70	0.01
I am constantly examined by nurses and doctors	22.67	1.63	0.77	28.12	1.85	0.54	-1.46	0.04
I hear the telephone ringing	23.13	1.63	0.71	27.69	1.85	0.67	-1.21	0.03
I am surrounded by unfamiliar machines	24.10	1.92	0.93	26.79	2.04	0.72	-0.69	<0.01

My blood pressure is checked several times a day	23.38	1.83	1.01	27.46	2.04	0.87	-1.05	0.02
IV drips are hanging over my head	19.75	1.63	0.82	30.81	2.23	0.59	-2.86**	0.17
Seeing family and friends only a few minutes a day	28.04	2.00	0.83	23.15	1.69	0.62	-1.30	0.03
Nurses and doctors talk too loudly	22.29	1.54	0.66	28.46	1.81	0.57	-1.68	0.06
I miss my husband or wife	22.98	1.96	0.91	27.83	2.23	0.76	-1.24	0.03
I do not know the doctor in charge of my care	23.73	1.79	0.83	27.13	1.96	0.66	-0.90	0.02
The nurse does not introduce herself to me	21.67	1.33	0.48	29.04	1.65	0.56	-2.05*	0.09

M—mean; SD—standard deviation; Z—test statistic; η^2—effect size index, * $p < 0.05$; ** $p < 0.01$; *** $p < 0.001$.
